# Autonomous demand, multiple equilibria and unemployment dynamics

**DOI:** 10.1007/s11403-020-00306-1

**Published:** 2020-10-31

**Authors:** Piero Ferri, Fabio Tramontana

**Affiliations:** 1 University of Bergamo, Bergamo, Italy; 2 Catholic University of the Sacred Heart, Milan, Italy

**Keywords:** Medium-run growth, Autonomous demand, Endogenous supply, Unemployment, Instability, The reconciliation process, Multiple equilibria, Simulations, E32, E12, J2, O40, C63

## Abstract

The paper presents a medium-run growth model driven by autonomous demand, where aggregate demand and supply interact and unemployment is present and plays different roles. In particular, it generates a feedback from supply to aggregate demand rooted in the presence of heterogeneous consumers and an uncertain environment. Two are the main consequences of this approach. The first is that multiple equilibria can be generated. The second is that equilibria may have different stability properties. In this perspective, growth becomes a dynamic process where initial conditions matter and history plays an important role.

## Introduction

There is no doubt that the Great Recession has stimulated the reconsideration of multiple equilibria in the literature. The idea of an economy having both “good” and “bad” equilibria has become appealing in almost every paradigm. So, while Geanakoplos (2003, 2010) stresses the importance of multiple equilibria characterized by the presence of agents with different degrees of liquidity within a General Equilibrium approach, Boissay et al. (2016) pursue the same objective by focusing on the banking crisis but within a DSGE model. In the same paradigm, Basu and Bundick (2017) study the relationship between uncertainty and multiple equilibria, while Aruoba and Schorfheide (2013) consider the presence of two equilibria in an economy marked by a zero lower bound in the rate of interest. Finally, Takahashi and Okada (2020), within an agent-based approach, present a dynamic model characterized by the presence of two equilibria based upon seller’s and buyer’s markets.

Also the Post-Keynesian field has been affected by the events of the economy, even though the presence of multiple equilibria has a long and persistent story (see Dutt 1997) rooted in the idea that growth is a history dependent phenomenon (see Robinson 1956). Many of the new contributions, above all those among the Kaleckian Post-Keynesians (KPK), have insisted on the role that income distribution can have in generating multiple equilibria within a growth model (see Assous and Dutt 2013).

In the present paper, we try to investigate another route, not necessarily an alternative one, leading to multiple equilibria by means of a nonlinear relationship between unemployment and durable consumption. The model represents an extension of two previous papers studying the relationship between autonomous demand, endogenous supply, growth and unemployment.

Fazzari et al. (2020) have inserted the role of autonomous demand into a Harrod–Minsky model, where aggregate supply accommodates to demand (on this aspect, see also Palley 2018). Unemployment remains bounded, while the reconciliation process between actual growth and the natural one takes place. Ferri et al. (2019) have enriched the model by taking into account a further feedback from unemployment to aggregate demand by considering heterogeneous consumers (see also Kaplan and Violante 2018), along with learning agents. In this environment, the dynamics between aggregate demand and supply become more interdependent and the instability process more complex (see also Ferri 2019).

In the present model, the role of multiple equilibria is stressed, so that the analysis of the triptych of properties, i.e., existence, uniqueness and stability, is completed. In particular, the emphasis is put on the demand for durable consumption goods, which is characterized by a trend component cyclically tempered by the (nonlinear) presence of the rate of unemployment. The consequences of this assumption are twofold. On the one hand, both the aggregate demand and supply determine the steady-state values of growth and unemployment. On the other hand, the presence of nonlinearity can generate two equilibria, one with high unemployment and low growth, and the other with low unemployment and high growth. The explanation of the two equilibria is rather simple: growth in the demand for durable consumer goods depends, among other things, on an exogenously given trend rate of growth of demand and the level of unemployment. Thus, there is a high unemployment with low growth equilibrium because of low aggregate demand growth due to low durable consumption growth, and a low unemployment high growth equilibrium because of high aggregate demand growth.

What is more, the two equilibria have different dynamic properties. The “virtuous” one is unstable, while the other one can be stable. The thesis put forward is that growth is a dynamic phenomenon drawn by disequilibrium processes that are history dependent. This state of affairs helps generating complex dynamics when considered within a global perspective and open the way to study policy measures in order to thwart their consequences. Furthermore, this conceptualization has an impact on the nature and on the dynamics of unemployment.

The structure of the paper is the following. Section 2 introduces autonomous demand into a medium-run model. Section 3 shows the determinants of aggregate demand. All variables are presented in intensive form, i.e., they are deflated by last period output. Section 4 introduces the specification of an accommodating supply. Section 5 considers the steady states of the model. Section 6 simulates the dynamics. Section 7 carries out a sensitivity analysis for various parameters. Section 8 discusses the basin of attraction of the global system. Section 9 concludes.

## Autonomous demand in a medium-run model

The strategic role of autonomous demand (see Hicks (1950) for a definition), which has been emphasized by Serrano and Freitas (2017) within the so called supermultiplier literature and which has been developed in a Kaleckian background (discussed by Lavoie 2016), has been criticized on both analytical and empirical grounds (see for instance Skott 2017). However, we agree with the justifications put forward by Dutt (2019), based upon both a deepening of the dichotomy between endogenous and exogenous variables, along with the relevance of the historical dimension. To these considerations, we add the hypothesis of a medium-run perspective, which is particularly appropriate to incorporate the role of autonomous demand.

According to Minsky (1982), the medium run, which is also called an intermediate period (see also Ferri 2011, 2019), is a historical period long enough to encompass the whole experience of an event such as the Great Depression. Roughly, it covers a decade. Each decade has almost always its historical characteristics. Three of them are worth considering. It is marked by a demand driver, which of course changes in time. This is the reason why autonomous demand can be represented by consumption durables, sustained by a particular monetary policy or by population growth. Or it may be represented by exports, as in the case of export-led economies. Also Government expenditures, as stressed by Allain (2015), can be mentioned. Finally, as underlined by Dutt (2019), autonomous demand needs not refer only to non-capacity creating expenditures. It might refer also to investment incorporating technical change.

In the medium run, investment affects capacity, and therefore, supply must enter into the picture. At the same time, there is uncertainty because the driver cannot work forever. These properties are incorporated in the present model, which is based on the same set up of Fazzari et al. (2020) and by Ferri et al. (2019) except for the consumption function, where its two components (see 3.2 and 3.5 below) are specified in a different way. In fact, the durable consumption function not only maintains the dependence on an exogenous rate of growth (g*), but it also depends, cyclically and nonlinearly, on the rate of unemployment. In this medium-run environment, consumption in durable goods is represented by the following equation:

2.1 $$ F_{t} = F_{t - 1} \frac{{\left( {1 + g^{*} } \right)}}{{\left( {1 + \lambda u_{t - 1} } \right)}} $$

where g* stands for the rate of growth of autonomous demand, while *u*_*t*_ represents the rate of unemployment. There has been a resistance to accept the presence of unemployment in the consumption function in a Keynesian context, the objection being that *Y* (output) and *u* (unemployment) are highly correlated. However, this criticism has underestimated the changes in the rules of the game underlying the labor market working. The inclusion of rate of unemployment helps considering two phenomena. On the one hand, it is a macro-variable that allows to introduce heterogeneity into the analysis. In fact, employed and unemployed are characterized by different propensities to consume (see also Kaplan and Violante 2018). On the other, it stands for a proxy of uncertainty characterizing non-insurable incomes. This is the line stressed by Carrol (1992), Malley and Moutos (1996) and Palley (2012). Both aspects are at the root of Eq. (3.5).

However, there is another aspect worth considering, i.e., the nonlinear role of the rate of unemployment. The effects of the Covid-19 on durables help justifying this assumption. It simply states that when unemployment reaches remarkable levels the welfare system becomes more ineffective in protecting the purchasing power of the unemployed. This circumstance, along with the greater uncertainty, contributes to negatively affect durable consumption.

The interaction between the two aspects generates a nonlinear relation, which generalizes the consumption function. In fact, it can deal also with extreme situations.

## Aggregate demand

The overall system, which tries to integrate aggregate demand and supply, consists of 10 equations in 10 unknowns. The system is nonlinear. Since we are interested in a global analysis, it will not be linearized. It follows that it will be studied mainly by means of simulations. In order to make the interpretation easier, the model is expressed in a recursive way.

The system is presented in an intensive form. In other words, each variable is divided through *Y*_*t*−1_, i.e., last period output.^1^ The model is represented in two blocks. The first group of equations mainly refers to the different component of aggregate demand, which are consumption (3.2), investment (3.3) and durables (3.5) and to their determinants, along with the expectation Eq. (3.1). In addition, there is Eq. (3.4) for the capital/output ratio (*v*_*t*_), which witnesses the feedback of investment on capacity.

3.1 $$ E_{t - 1} g_{t} = (1 - \alpha )g_{t - 1} + aE_{t - 2} g_{t - 1} $$

3.2 $$ c_{t} = c_{1} \left( {1 + E_{t - 1} g_{t} } \right) $$

3.3 $$ i_{t} = \delta v_{t} + Eg_{t} v_{t} + \beta \left[ {v^{*} (1 + E_{t - 1} g_{t} )^{2} + (1 + Eg_{t} )v_{t} } \right] $$

3.4 $$ v_{t} = \frac{{v_{t - 1} (1 - \delta )}}{{(1 + g_{t - 1} )}} + \frac{{i_{t - 1} }}{{(1 + g_{t - 1} )}} $$

3.5 $$ f_{t} = \frac{{f_{t - 1} }}{{\left( {1 + g_{t - 1} } \right)}}\left( {1 + \frac{{g^{*} }}{{1 + \lambda u_{t - 1} }}} \right) $$

3.6 $$ g_{t} = c_{t} + i_{t} + f_{t} - 1 $$

Equation (3.4) is a definition, (3.6) an equilibrium condition, while the remaining ones are behavioral equations.

Equation (3.1) supposes that expectations of the rate of growth (*g*_*t*_) are formed according to an adaptive rule (see Ferri et al. 2019, for more sophisticated formulae based upon learning). Equations (3.2) and (3.5) represent consumption in non-durable and durable goods, respectively. The former is the simple Keynesian consumption function expressed in terms of expectations, while the latter is Eq. (2.1) normalized by *Y*_*t−*1_.

Equation (3.3) introduces the investment function, based upon capital adjustment. In other words, investment adjusts according to the gap existing between expected capital, based upon an expected demand growth, and the existing stock of capital. In this perspective, the normal capital/output ratio v* and growth expectations become the strategic variables.^2^ Equation (3.3) plays different roles: (i) it replaces depreciation (*δ*); (ii) it accounts for steady-state growth in desired capacity; and (iii) at least partially (according to the value of *β*) it closes the gap between actual and desired capacity, where v* is the normal capital/output coefficient.

Equation (3.4) expresses in intensive form the accumulation equation, which depends on depreciation and last period investment. It shows the evolution of the capital/output ratio derived from the accumulation equation vis-à-vis the normal v*. These two variables refer to different level of income, being *Y*_*t−*1_ for *v*_*t*_ and *Y*_*t*_ for v*.

Equation (3.6) represents the equilibrium in the product market, which generates the Harrod’s warranted rate of growth.

## An endogenous natural rate of growth

According to Harrod (1939), the natural rate of growth can be approximated by the rate of growth of productivity and labor supply. In recent times, many authors have tried to endogenize this variable (see Dutt 2010; Lavoie 2016, for a synthesis). The peculiarity of the present analysis is that both components are endogenized, as appears from this second block of equations.

4.1 $$ \tau_{t} = \theta_{0} + \theta_{1} \frac{{i_{t - 1} }}{{v_{t - 1} }} $$

4.2 $$ gN_{t}^{s} = \rho_{0} - \rho_{1} u_{t - 1} $$

4.3 $$ gN_{t} = \frac{{\left( {1 + g_{t} } \right)}}{{\left( {1 + \tau_{t} } \right)}} - 1 $$

4.4 $$ u_{t} = 1 - \left( {1 - u_{t - 1} } \right)\frac{{\left( {1 + gN_{t} } \right)}}{{\left( {1 + g_{t}^{s} } \right)}} $$

A Leontief production function is at the root of the labor market considered, along with some dynamic laws concerning productivity and labor supply. In particular, Eq. (4.1) refers to the rate of growth of productivity (*τ*_*t*_). It depends on capital accumulation as Kaldor (1978) has stressed long-time ago (see also McCombie 2002) and as the recent literature on investment on R&D has confirmed.^3^

Equation (4.2) represents the rate of growth of labor supply (*gN*_*t*_^*s*^) that depends negatively on the rate of unemployment, as the recent experience has shown (see Delong and Summers, 2012). Equation (4.3) generates the rate of growth of labor demand (*gN*_*t*_), while Eq. (4.4) defines the rate of unemployment (*u*_*t*_) in terms of the rate of growth of its two determinants.

The 10 equation system has the following 10 unknowns:

*E*_*t*−1_*g*_*t*_, *c*_*t*_, *f*_*t*_, *i*_*t*_, *v*_*t*_, *g*_*t*_, *gN*_*t*_, *gN*_*t*_^*s*^, *u*_*t*_ and $\tau_{\text{t}}$, given the exogenous variables v* and g*.

## Steady states

A medium-run analysis is more interested in considering the average rates of growth than steady states. However, since in the present model, the emphasis is on multiple equilibria a discussion on steady state is worth doing even though they might not be ever reached.

In steady state, the following relationships hold:

5.1 $$ i_{0} = v_{0} \left( {g_{0} + \delta } \right) $$

5.2 $$ c_{0} = c_{1} \left( {1 + g_{0} } \right) $$

5.3 $$ f_{0} = 1 + g_{0} - c_{0} - i_{0} $$

5.4 $$ v_{0} = v^{*} \left( {1 + g_{0} } \right) $$

where the values are obtained by the respective equations, while (5.3) is obtained through (3.6). Equations (3.5) generates:

5.5 $$ g_{0} = \frac{{g^{*} }}{{1 + \lambda u_{0} }} $$

Since the natural rate of growth can be expressed by the following equation:

5.6 $$ g_{t}^{s} = (1 + \tau_{t} )(1 + gN_{t}^{s} ) - 1 $$

**Fig. 1:**
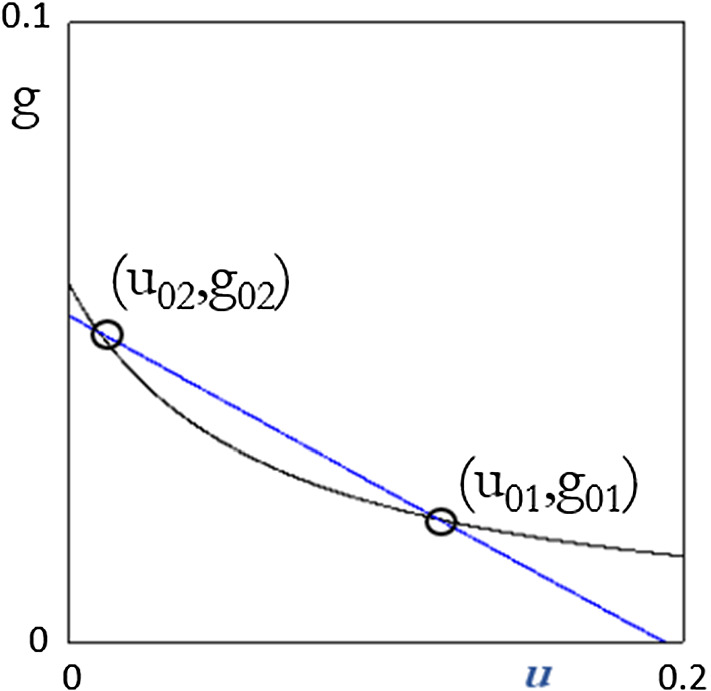
Multiple equilibria of the system. The black curve represents Eq. (5.5), while the blue one is obtained from Eq. (5.6)

Considering the steady-state value of its component, along with (5.5), the following picture can be obtained (Fig. 1).

Where parameters are chosen as specified in Table 1.^4^

**Table 1:** The values of the parameters

*c*_1_ = 0.608	*δ* = 0.12	v* = 0.6505
*λ* = 15.7895	g* = 0.0579	*β* = 0.38
*α* = 0.69	*ρ*_0_ = 0.0212	*ρ*_1_ = 0.2252
	*θ*_0_ = 0.005	*θ*_1_ = 0.15

The system is characterized by two equilibria, one low and the other high, but both economically feasible. The slopes of the two curves are different at the two equilibrium points. However, this does not allow to draw conclusions about their stability. This depends crucially on the dynamic analysis. For instance, we will see that the adaptive expectations parameter *α* has no role in shaping the two curves (i.e., it does not influence the equilibrium values), but it plays an important role in their stability.

In order to prove the robustness of the result about the existence of multiple equilibria, the algebraic value of the equilibrium rates of unemployment is to be recovered. By equating (4.2) to (4.3), the following second degree equation is found for *u*_0_:

5.7 $$ Au_{0}^{2} - Bu_{0} + C = 0 $$

where

$$ \begin{aligned} A & = \rho_{1} + \theta_{1} \lambda g* \\ B & = \rho_{1} \left[ {1 + \theta_{0} + \theta_{1} \left( {g* + \delta } \right) + \lambda g* \left[ {\left( {1 + \rho_{0} } \right)\rho_{1} \theta_{1} - 1} \right]} \right] \\ C & = \left( {1 + \rho_{0} } \right)\left[ {1 + \theta_{0} + \theta_{1} \left( {g* + \delta } \right)} \right] - \left( {1 + g*} \right) \\ \end{aligned} $$

For the values of the parameters illustrated in Table 1, one obtains two roots, *u*_01_ and *u*_02_, which are both positive and less than 1. They are both feasible, and therefore, the model generates multiple equilibria that are economically feasible and meaningful.

It follows that the rate of unemployment depends on both aggregate demand and supply aspects. It represents a generalization of Harrod’s natural rate, because the unemployment rate reconciles the product market equilibrium with the labor market one. It differs from Friedman (1968)’s definition of natural rate which is rooted in Walrasian economics. It also differs from Malinvaud (1977)’s disequilibrium analysis where demand and supply aspects are not simultaneously present.

Equation (5.7) admits two roots as long as its discriminant (∆) is strictly positive, that is:

5.8 $$ \Delta = B^{2} - 4AC > 0 $$

which cannot be easily solved, but considering the not negativity of the squared term (*B*^2^), a sufficient (but not necessary) condition for the existence of two equilibria is related to the strict positivity of the second addendum:

$$ - 4AC > 0\mathop \Leftrightarrow \limits_{{}} C < 0 $$

Where we have taken into account the restrictions about the values each parameter may assume, implying the positivity of *A*. The last inequality permits to identify a threshold value ($\tilde{g}$) for the exogenous rate of growth:

5.9 $$ g^{*} > \tilde{g}  \,\,{\text{where}}\,\,\tilde{g} = \frac{{\left( {1 + \rho_{0} } \right)\left( {\theta_{0} + \theta_{1} } \right) + \rho_{0} }}{{1 - \left( {1 + \rho_{0} } \right)\theta_{1} }} $$

So, for $g^{*} < \tilde{g}$ the systems does not admit any equilibrium, while for $g^{*} > \tilde{g}$ we have two equilibria.^5^ For the values of the parameters illustrated in Table 1, one obtains two roots, u_01_ and u_02_, which are both positive and less than 1. They are both feasible and therefore the model generates multiple equilibria that are economically feasible and meaningful. The threshold value of the rate of growth in fact is $\tilde{g} \simeq 0.05277$, and it is lower than g^*^. Keeping into consideration that condition (5.9) is a sufficient but not necessary condition for the coexistence of two equilibria, we can easily state that for a large region of the parameter space around the values of Table 1, two equilibria coexist.

## Simulating the dynamics

The above nonlinear system has been simulated. The values of the parameters are still those written in Table 1. Given the values of the parameters, the steady-state values for *g* and *u* are illustrated in Table 2.

**Table 2:** The steady-state values of *g* and *u*

	Bad equilibrium	Virtuous equilibrium
Rate of growth	*g*_01_ = 0.02	*g*_02_ = 0.05
Rate of unemployment	*u*_01_ = 0.12	*u*_02_ = 0.01

**Fig. 2:**
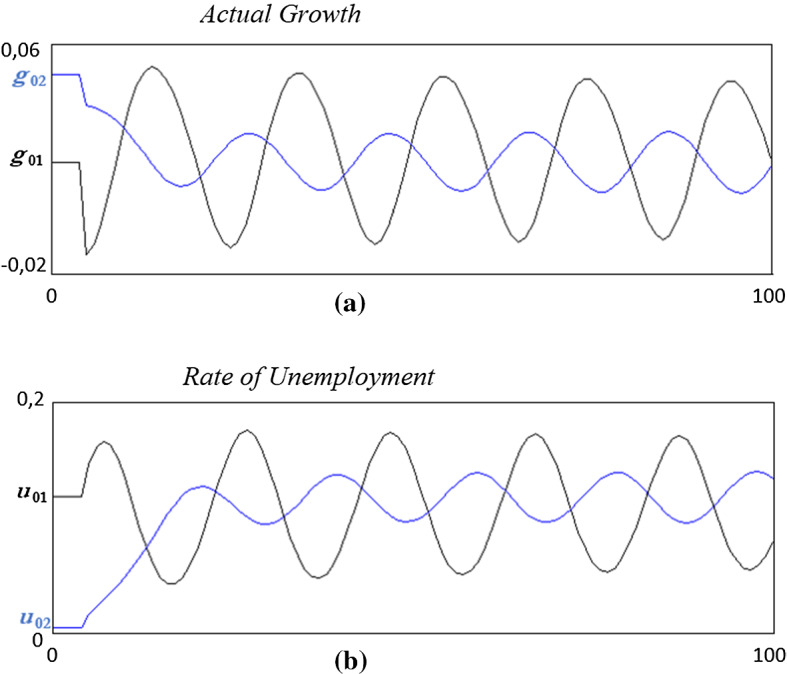
The dynamics with multiple equilibria

In order to set the dynamics in motion, both the steady states of the model have been shocked at period 5.^6^ The ensuing dynamics are illustrated in Fig. 2.

Some observations are worth stressing. The first is that the system fluctuates and remains bounded around the steady state with high unemployment (and low actual growth), which may be denoted as *bad regime*. The second is that the model is robust to changes in the period length. In fact, the length is 100 periods by it can expanded to 10.000, the only difference is that the fluctuations of the trajectory coming from a perturbation of the steady state with low unemployment (*good regime*) will gradually converge to the amplitude on the fluctuations obtained starting from the high unemployment steady state. The third is that the dynamics concern both equilibria even though they end up in the bad regime. It follows that a global dynamic analysis is necessary. Moreover, the timeplots suggest that the bad regime equilibrium for these parameters’ values is an unstable focus, surrounded by a stable closed invariant orbit. So, by varying the values of the parameters fluctuations may become of higher/smaller amplitude, can diverge of converge to the steady state if it becomes a stable focus. The role of the various parameters will be investigated in the next section.

## Sensitivity analysis

**Fig. 3:**
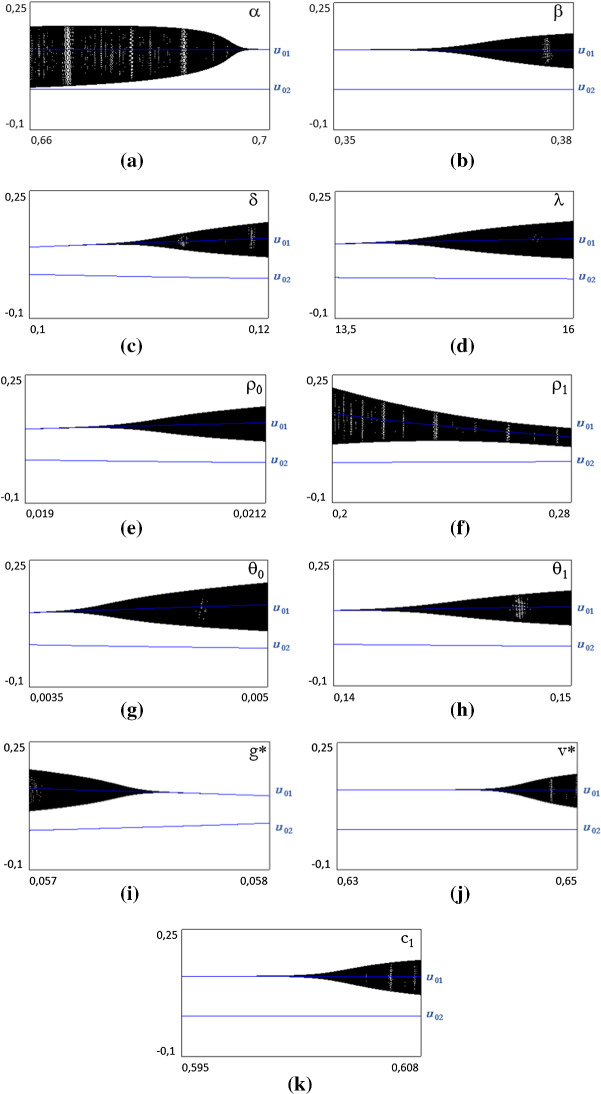
Bifurcation diagrams for the various parameters. In each diagram, all the other parameters are fixed at Table 1 values

Figure 3a–k presents various exercises of sensitivity analysis referred to the various parameters. Numerical analysis suggests that by varying the value of the parameters, a supercritical Neimark–Sacker (NS) bifurcation of the bad regime steady state may occur, creating bounded and persistent fluctuations.^7^

Let us summarize the results by referring to the different groups of parameters. The first group refers to the canonical Harrodian parameters: v*, *δ* and *c*_1_, which confirm the usual role of inducing instability at their increasing.

The second group refers to parameters derived from small changes to the canonical model: g*, *λ*, *β*. Also in this case, they behave in conformity to analytical expectations. While the first one is stabilizing, the other two tend to be destabilizing. The third group only includes *α*, which refers to expectations, which stabilizes the system. Even though the present specification of the expectation function may raise perplexities, Ferri et al. (2019) have shown how the dynamics of the model is robust to changes in the specification of expectations, above all those based upon some form of learning.

Finally, the last group of parameters refers to supply: *θ*_0_, *θ*_1_, *ρ*_0_ and *ρ*_1_. The first two refer to productivity, while the second refer to labor supply. In the present model, only *ρ*_1_ is apparently stabilizing. The results differ from the previous models, like Fazzari et al. (2020). In fact, while in the former the matrix of the linearized model is recursive, implying that the supply parameters have only an impact on the amplitude of the fluctuations but not on their stability, in the latter aggregate demand and supply are interdependent, because of the specification of consumption function. In the present model, this interdependence is obtained in the demand for durable, where the rate of unemployment acts as a contrasting force to the driver g* and creates two equilibria with opposing dynamic properties. It follows that only a faster reaction of the labor supply (by means of an increase in *ρ*_1_) is stabilizing, while in all other cases, the opposite situation occurs.

## A sketch of global analysis

Even though the characteristics of each equilibrium are important^8^ to be considered, there is no doubt that the forces underlying the dynamics of the entire system can only be grasped by a global analysis, which can deal with different initial conditions.

**Fig. 4:**
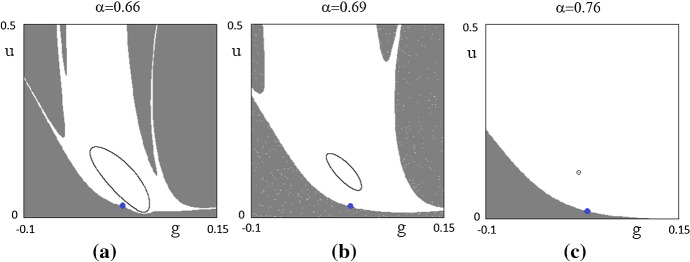
Basins of attraction. The basin in white denotes initial conditions of g and u leading to the stable equilibrium (**c**) or to the closed invariant orbit (**a**, **b**). The virtuous equilibrium is the point in blue. The initial conditions of the other variables are kept fixed at their steady-state values

In order to study the global properties of the model, the identification of the basins of attraction becomes a fundamental task. It is worth stressing that it can only be obtained by referring to the nonlinear formulation of the model. To this purpose, Fig. 4a–c is worth considering.

All the parameters are fixed (see Table 1) and in the plane (*g*, *u*) the attractor (the curve) can be identified. The white zone represents all the initial conditions leading to a convergence toward the bad regime steady state (panel c) or toward the curve (panels a and b), implying the presence of persistent fluctuations. The gray zone leads to divergent fluctuations. The (unstable) virtuous equilibrium is the blue point. So, by starting with a value of *α* such that the steady state is locally stable (panel c), the value of *α* is reduced and the steady state undergoes a NS bifurcation creating a closed invariant orbit around it (panel b). By continuing to decrease the value of *α*, the curve becomes larger (panel c) and fluctuations are characterized by a higher amplitude. In panel c, we can also see that the curve appears quite close to have a contact with the border of the basin of attraction. When it occurs every trajectory diverges (*final bifurcation*). Similar numerical results can be obtained by using the other parameters, increasing or decreasing them according to their stabilizing or destabilizing role, as previously analyzed.

**Fig. 5:**
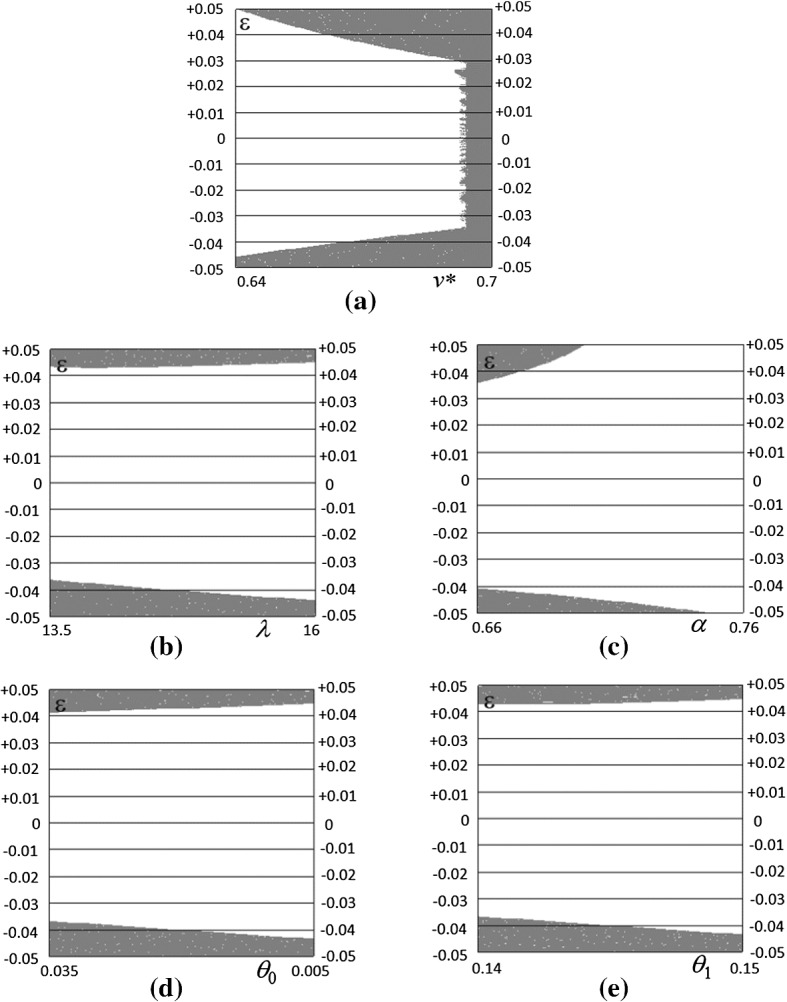
Robustness to different initial conditions. Points in white denote combinations of parameter (on the *x*-axis) and different initial conditions on *Eg* (on the *y*-axis) leading to convergent fluctuations, while gray points denote combinations of parameter values and initial conditions leading to diverging fluctuations

In order to identify the shape of the basins of attraction, it is important also to know how different the initial conditions must be in order to move the system from bounded fluctuations to diverging ones. In Fig. 5a–e, we have drawn combinations of parameters and initial conditions values leading to an absorption of fluctuations (white points) or to diverging fluctuations (gray points). Different initial conditions have been applied to *Eg* and the other parameters are fixed as in Table 1.

Two observations are worth doing. The first is that the latter picture can be reproduced for all the other parameters.^9^ The other is that the basin of attraction shows that the dynamics of the model is rather robust.

## Concluding remarks

The present paper has analyzed a medium-run model of growth, driven by autonomous demand, where aggregate demand and supply interact and where unemployment plays a prominent role. In particular, the paper takes into account a demand for durables characterized by a nonlinear relationship between an autonomous component, driven by an exogenous rate of growth g*, and the rate of unemployment. This enrichment does not alter the strategic role of the autonomous component, but it allows to obtain multiple equilibria capable of generating a more complex dynamic analysis.

In our model, persistent fluctuations are generated and this is the result of the interplay between aggregate demand and supply. Furthermore, fluctuations may be bounded for meaningful values of the parameters and this allows to generate a value of the rate of unemployment that is economically viable. Furthermore, a particular methodological lesson can be learned from the present model with multiple equilibria. The medium-run growth is a dynamic result of the interaction between aggregate demand, an accommodating supply and the role of drivers and constraints. In this context, history matters. The effective rate growth is the result of the relative permanence in the areas delimited by the two equilibria and this depends on the initial conditions.

The model can be extended in several ways. First, the working of the labor market can be enriched by introducing the analysis of flows. In the second place, the role of constraints can be deepened (see Dupraz et al. 2019). The constraints imposed by unemployment may not be operative only in steady state, but they can become effective during the disequilibrium dynamics. In these cases, reaching an unemployment barrier implies approximating a production ceiling and therefore prices cannot be supposed to be given. Rationing must be replaced by the working of prices. However, in this case, a regime switching analysis can be carried out (see Ferri and Tramontana 2017; Ferri 2019). Thirdly, in order to have a fully integrated model, also financial aspects are to be considered and these are particularly important when heterogeneous agents are considered. Finally, the perspective of the model can be extended. Beyond the medium run, there are situations where both structures (see Pasinetti 1981) and institutions change and where new dynamic methodologies are called for (see, for instance, Dosi et al. 2012).

## Footnotes

1 The KPK approach, being focused on the long run, divides through *K*.

2 In other words, $I_{t} = K_{t + 1}^{*} - \left( {1 - \delta } \right)K_{t - 1} = v*\left( {1 + E_{t - 1} g_{t} } \right)^{2} - \left( {1 - \delta } \right)K_{t}$, where expectations are based upon information available at time *t* − 1. Normalizing this equation and by adding and subtracting *Eg*_*t*_*v*_*t*_, (3.3) in the text is obtained.

3 An exogenous explanation is put forward by Dey (2017). In Fazzari et al. (2020), also the rate of unemployment is present, following a Hicksian intuition (see Hicks 1932). However, in this context this variable has been dropped because it would complicate the nonlinearity of the system.

4 The parameters are similar to those used in Fazzari et al. (2020) in Table 1. They are justified from an economic point of view in a Supplementary Appendix. As far as λ is concerned, it has been calibrated in such a way to generate meaningful values of *g* and *u*.

5 Technically speaking, we can say that at $g^{*} = \tilde{g}$ a *Fold* bifurcation occurs, because there is a passage from *n* to *n *+ 2 equilibria (in this case from 0 to 2).

6 We used an additive shock of + 0.1 on the actual growth, only at *t* = 5. The same result can be obtained by directly starting with an initial condition of the system closed, but not exactly coincident, with the steady states.

7 The further conditions necessary to study their stability are discussed by Kuznetsov (2004). See also Agliari et al. (2005).

8 A linearized version of the model shows that the modulus of the complex roots is, respectively, 0.87 for the bad equilibrium and 1.24 for the virtuous one.

9 Fazzari et al. (2020) have shown that the value of *v** can be increased without creating instability in the presence of an adjustment process in this variable.
